# The Relationship between Working Memory and Arithmetic in Primary School Children: A Meta-Analysis

**DOI:** 10.3390/brainsci13010022

**Published:** 2022-12-22

**Authors:** Yuxin Zhang, Andrew Tolmie, Rebecca Gordon

**Affiliations:** UCL Institute of Education, London WC1H 0AL, UK

**Keywords:** working memory, arithmetic, working memory domain, operation type, mental arithmetic, written arithmetic, meta-analysis, visuospatial working memory, verbal working memory

## Abstract

Working memory (WM) plays a crucial role in the development of arithmetic ability. However, research findings related to which factors influence the relationship between WM and arithmetic skills are inconsistent. The present meta-analysis aimed to examine the links between WM and arithmetic in primary school children and investigate whether this is dependent on WM domains (i.e., verbal, visual, spatial), child age, arithmetic operation type, and arithmetic task type. A total of 11,224 participants with an age range of 6- to 12 years, from 55 independent samples were included in the meta-analysis. Analysis of 46 studies with 187 effect sizes revealed an overall significant and medium correlation between WM and arithmetic. Heterogeneity analyses indicated that verbal WM showed a stronger correlation with arithmetic than visuospatial WM, and that correlations between verbal WM and arithmetic declined with age, whereas correlations between spatial-sequential, and spatial-simultaneous WM and arithmetic remained stable throughout development. Addition and subtraction were more involved in verbal WM than multiplication and division. Moreover, mental and written arithmetic showed comparable correlations with WM in all domains. These findings suggest moderation effects of WM domains, age, and operation types in the WM-arithmetic relationship and highlight the significant role of verbal WM in arithmetic ability in primary school children.

## 1. Introduction

There is considerable evidence for an important role of working memory (WM) in the development of arithmetic ability (for reviews, see [1,2,3]). WM is commonly defined as an attention-based system with a limited capacity to temporarily store and manipulate information in pursuit of a specific goal [4,5]. To solve calculation problems (e.g., 47 + 9 or 23 − 6), children need to execute a series of steps, including recalling math facts from long-term memory, borrowing or carrying, splitting the problem into subproblems, switching between different operations, and tracking intermediate solutions while manipulating other information [6]. All of this necessitates a large amount of information storage and concurrent processing, which makes sense given the research evidence that implicates WM in this process of monitoring and coordinating thefse activities [6]. There is also evidence for the predictive potential of WM for later arithmetic performance [7,8,9,10]; and further evidence of children with dyscalculia, exhibiting WM deficits [11]. Furthermore, neuroimaging studies have demonstrated an overlap of brain activation areas (e.g., parietal and pre-frontal cortex) during WM tasks and arithmetic tasks [12].

Although these studies present useful information on the critical role of WM in arithmetic, there is lack of agreement on the WM-arithmetic relationship across WM domains (i.e., verbal WM and visuospatial WM). Some studies have indicated that verbal and visuospatial WM play distinct roles in arithmetic. Szűcs et al. [13] suggested a robust predictive role of visuospatial WM but not verbal WM in arithmetic performance of 9-year-old children. Andersson and Östergren [14] demonstrated a visuospatial WM deficit (not a verbal WM deficit) in children with math learning difficulties who performed poorly on arithmetic tasks. In contrast, Seitz and Schumann-Hengsteler [15] reported a critical role of verbal components of WM but not visuospatial ones in mental addition and multiplication. Whereas others reported that verbal WM and visuospatial WM have associations of a similar magnitude with arithmetic [16,17]. Furthermore, some studies have shown a shift from reliance on visuospatial WM to verbal WM in arithmetic throughout development, with a reliance on visuospatial WM diminishing with age; and links with verbal WM increasing [18,19,20].

It has been posited that younger children, compared to older children, rely more on visuospatial mental representations and strategies when performing calculations, such as employing mental number lines and counting with concrete objects [21]. It is argued that, as children grow older, with experience accumulated through practice, they can then consolidate and retrieve verbal number facts and operations from long-term memory., thus drawing more on verbal WM [22]. Similarly, when learning new skills, children frequently employ procedural strategies (e.g., counting and deconstruction), which may depend largely on visuospatial WM [23], but when familiar with arithmetic, children might use verbal WM for fact retrieval and maintenance [24]. Additionally, as children’s language skills develop, verbal codes and strategies, such as preserving numerical information through rehearsal, appear to become increasingly efficient [9].

However, other studies have reported a converse pattern in terms of developmental changes in the links between arithmetic and WM domain. Meyer et al. [25] and Soltanlou et al. [26] found that in primary school children, verbal WM predicted arithmetic performance at an early stage, but that visuospatial WM predicted arithmetic ability in subsequent grades. This might be explained if it is considered that, at the beginning of school, learning is largely based on verbal codes to retain information and retrieve knowledge from memory [27]. Given the meaning of numerical symbols relate to their spatial arrangement, as the mental representation system becomes more sophisticated, older children might use more abstract, visually oriented decoding of equations and retrieval of number facts [28,29]. For instance, when calculating 32 − 17, using spatial position in the mental model could help children map quantitative relations to the numerical symbols (e.g., 2 is less than 7; “borrow a 1 from the 10 s, etc.).

Further to the contrary findings described here, some studies have found that the relationship between WM domain and arithmetic does not vary with age. An experimental study revealed a similar effect of WM load (both verbal and visuospatial domains) on the accuracy and speed of arithmetic performance in all age groups from 9-year-olds to adulthood [23]. In terms of younger age groups, Vieira, Ribeiro, Farias and Freitas [16] found significant and moderate correlations between arithmetic and verbal and visuospatial WM from aged 6–11 years. Furthermore, Allen et al. [30] found a substantial effect of visuospatial WM on mathematical performance (including arithmetic) in children aged 0–16 in a meta-analysis, unaffected by age.

Given these conflicting findings, it is worthwhile considering which factors might influence these differing outcomes beyond age and WM domain. With regard to WM domain, some studies have proposed a further subdivision of visuospatial WM based on WM task type. These are: (1) visual tasks, recalling information about object properties (e.g., shapes and colours); and (2) spatial tasks, recalling information about location [31]. The dissociation of visual and spatial WM has been supported by evidence from studies with children aged 6–10, which have identified different relationships with arithmetic based on these different WM task types [32,33]. For example, Fanari et al. [33] found spatial WM to be linked to basic numeracy at the age of 6.5 years, and that addition and subtraction were best predicted by both spatial and visual WM at the age of 7.5 years. However, Holmes, Adams and Hamilton [32] found spatial WM predicted mathematic performance (including 4 types of arithmetic operations) in 7- to 8-year-old children, while visual WM was relied upon at the age of 9 to 10.

Spatial WM tasks have been further sub-classified based on how information is presented. These are: (1) spatial-simultaneous tasks, which require memorizing an array of spatial locations that are presented simultaneously; and (2) spatial-sequential tasks, which require recalling a sequence of spatial locations presented in a specific order [34]. Recent findings indicated separate roles of spatial-sequential and spatial-simultaneous WM in arithmetic [35,36]; whereby spatial-simultaneous WM in 7-year-old children predicted concurrent mathematics performance [37], and spatial-sequential WM predicted mathematics performance two years later [35]. However, it should be noted that a systematic review by Allen, Higgins and Adams [30] with typically developing populations found no difference in correlations with mathematics between spatial-simultaneous and spatial-sequential WM.

The research discussed here implies that different WM domains and sub-domains hold differing relationships with arithmetic that might, partly, be dependent on age. However, findings from these studies examining this finer grain of WM domain across age groups are variable. In addition, the relatively small sample sizes within these studies (e.g., [11], *n* = 4) might be a factor affecting the possibility of detecting (or not detecting) actual effects, or resulting in less precise estimations of effects (e.g., increasing the margin of errors) due to the large variance [38]. One solution is to synthesize the evidence to gain a view of the overall effects observed in the studies examining this WM-arithmetic relationship.

Another influencing factor to consider is the specific operation (i.e., addition, subtraction, multiplication, division) within the arithmetic sums used in these studies to measure arithmetic ability. For example, studies using dual-task experiments have reported interactions between WM domains and specific arithmetic operations suggesting a unique contribution of visuospatial processes to subtraction and verbal processes to multiplication [39,40,41]. Furthermore, it has been suggested that addition and subtraction rely on visualising the manipulations of numbers, whereas multiplication and division are more based on the retrieval and maintenance of memorized verbal facts [19].

Related to this, Demir, Prado and Booth [12] provided neuropsychological evidence from 9- to 12-year-old children indicating that higher spatial WM was related to enhanced activation in the right intraparietal sulcus during both multiplication and subtraction while the association between higher verbal WM and enhanced activation in the left middle temporal gyrus was merely found in multiplication. However, a large behavioural study involving 22731 elementary school students identified significant and comparable correlations between visuospatial WM and all four operations (i.e., addition, subtraction, multiplication, and division) [19]. Similarly, Van de Weijer-Bergsma, Kroesbergen and Van Luit [20] detected no differences in the influence of verbal and visuospatial WM on these operations in children aged 7–12.

Owing to the variability in findings discussed thus far, it is not yet possible to draw conclusions on the relationship between WM and these four operations. The paucity of research on direct comparisons of the WM-arithmetic relationship across WM domains and operation types (but see Van de Weijer-Bergsma, Kroesbergen and Van Luit [20]) may contribute to the inconsistency in findings. Therefore, the current study explored the influence of operation types on the WM-arithmetic relationship systematically by aggregating data from published studies.

The final consideration in this meta-analysis with regard to which factors might be influencing the variability in findings from studies examining the WM-arithmetic link is arithmetic task type. Both mental and written arithmetic tasks have been used to measure arithmetic ability as an outcome as explained by WM ability. However, neuroimaging studies point to different brain regions activated during mental, compared to written, arithmetic, suggesting these are quite separable abilities. For example, activation in the anterior superior longitudinal fasciculus (associated with visuospatial WM that subserves spatial attention control and visual process) has been linked to performance on mental arithmetic tasks but not on written arithmetic tasks [42,43]. There are several explanations as to why mental arithmetic is thought to rely more on general WM resources than written arithmetic [2,44,45] For example, when calculating on paper, children can pause in the execution of processes, note down interim outcomes, and so are not forced to hold all information in mind, leading to fewer demands on WM resources [46]. In contrast, during mental arithmetic, children are expected to concurrently store items, retrieve number facts from long-term memory, and manipulate numbers while preserving intermediate values in their minds, which presumably demands greater WM resources [47]. However, a study by Allen and Dowker [48] assessed visuospatial WM, mental arithmetic, and written arithmetic in 36 children aged 6- to 7-years old, and found that both mental arithmetic (*r* = 0.56) and written arithmetic (*r* = 0.60) were similarly correlated with visuospatial WM.

The evidence presented here suggests that variability in findings related to the WM-arithmetic relationship might be influenced by arithmetic task type. Therefore, in order to provide a comprehensive analysis of the research in this field, the current study aimed to conduct a meta-analysis to examine whether the types of arithmetic tasks affect the relationship between WM and arithmetic, whilst also considering WM domain, sub-domains, age and operation type. The research questions were:

(1) Is there a significant relationship between WM and arithmetic?
(2) Is the WM-arithmetic relationship different dependent on types of WM domains, WM sub-domains, children’s age, arithmetic operations, and types of arithmetic tasks?

## 2. Materials and Methods

### 2.1. Search Strategy and Study Selection Criteria

**Figure 1:**
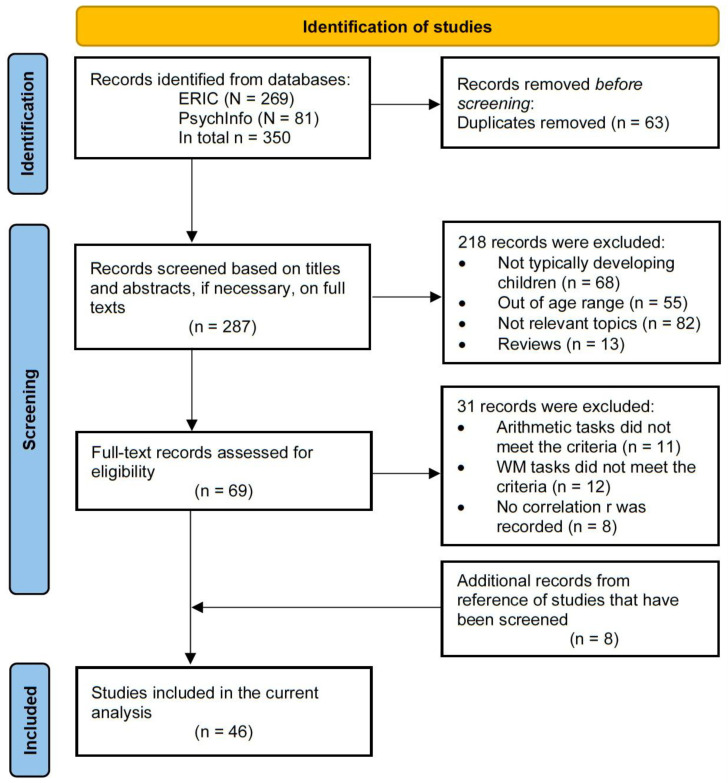
The PRISMA flow chart of literature search and screening process.

The meta-analysis was conducted and reported according to the Preferred Reporting Items for Systematic Reviews and Meta-Analyses (PRISMA) guidelines [49]. The identification and screening process is outlined in Figure 1. A search of peer-reviewed journal articles from 2000 through July 2022 was conducted in the Education Resources Information Centre (ERIC) and PsycInfo. These two databases were chosen since they provide trusted indices and the widest access to relevant literature in education and psychological science.

The search was conducted with combinations of the following terms: “working memory” AND (“arithmetic” OR “calculation” OR “computation”). This search was run over a period of weeks, with the last wave of new articles identified on 30 July 2022. The initial search yielded 269 results from ERIC and 81 results from PsychInfo. After removing duplicates (*n* = 63), 287 studies were screened in two stages. At stage 1, titles and abstracts were reviewed and studies that were obviously out of scope using four inclusion criteria were removed from further analysis: (1) The language of publication was English; (2) Participants included typically developing populations only: (3) Participants were elementary school children (aged 6 to 12); (4) The research was related but not limited to cognitive development and math learning; and, (5) The paper was an empirical study (i.e., not a review, meta-analysis or systematic review). This initial screening excluded 218 studies, 68 on atypically developing populations, 55 with participants outside the age range, 82 on irrelevant topics, and 13 reviews. This resulted in 69 remaining studies (see Supplementary File S2).

At stage 2, full texts of the remaining studies were evaluated in terms of their suitability for the meta-analysis, based on the following criteria. First, the study had to include at least one quantitative measure of working memory that involved processing and maintaining information simultaneously. Studies in which WM tasks only assessed memory capacity without a processing manipulation (e.g., forward digit span task) were excluded. Similarly, studies where WM tasks assessed other general cognitive skills such as inhibitory control and cognitive flexibility rather than working memory were excluded. Second, the study had to include at least one quantitative measure of arithmetic ability that involved performing exact calculations (not approximate estimation) such as “6 + 5 = ?” or verifying whether a given arithmetic equation such as “6 + 5 = 13” was correct or not. Studies using word problems (e.g., There are 96 passengers in the first bus and 107 passengers in the second bus. How many passengers are there in total?) were excluded to ensure language comprehension demands across arithmetic tasks were as low to minimize the influence of individual variation in reading ability on findings from the studies. Arithmetic performance also had to be measured in terms of accuracy; studies using reaction time only were excluded. Third, the study had to report at least one direct correlation (i.e., *r,* not partial correlation) between WM and arithmetic. Both concurrent and longitudinal correlations were included.

Where studies did not provide sufficient information to evaluate them, the authors were contacted for the required information based on the inclusion and exclusion criteria. Of the 69 studies, 31 were excluded during the full-text screening, of which 12 did not meet the criteria for working memory tasks, 11 did not meet the criteria for arithmetic tasks, and 8 were excluded due to the lack of correlation data (*r*) between working memory and arithmetic. Additionally, 8 more eligible studies were identified through a search of the reference sections of studies that have been screened. In summary, a total of 46 studies were included in the meta-analysis.

### 2.2. Data Extraction and Coding

The following information was coded for analysis: (a) the mean age of the sample; (b) descriptions of working memory and arithmetic tasks; (c) the working memory domain (i.e., verbal WM, visuospatial WM, and composite WM) measured; (d) the subtype of spatial working memory (i.e., spatial-sequential WM and spatial-simultaneous WM); (e) the operation type (i.e., addition, subtraction, multiplication, and division); (f) the arithmetic task type (i.e., mental arithmetic and written arithmetic); (g) the correlation *r* between WM and arithmetic performance; and (h) the sample size for each *r*. In addition, the first author, publication year, time lag between the measurement of WM and arithmetic, test environment (i.e., individual test vs. group test), and the research area were coded as descriptors. Descriptions and examples of WM domains, subtypes of spatial WM, and arithmetic task types are shown in Table 1. Key characteristics of studies included in this analysis are outlined in Supplementary File S1.

### 2.3. Analytical Strategy

The effect sizes were indexed by parametric *r* values between WM and arithmetic. To normalise the distribution of the correlation coefficients, each *r* was converted to Fisher’s Z_r_ which was then used in all analyses. The *r*-to-z conversion leads to more precise estimation of effect sizes when performing meta-analysis [61]. The results (i.e., the effect and its confidence interval (CI)) were translated back to standard correlation coefficients for reporting as *r* is more easily interpretable. It was anticipated that the effect sizes collected were sampled from a distribution of effect sizes rather than a common effect size across studies. Substantial between-study heterogeneity was also anticipated, with some variations assumed to be caused by methodology within each study included in the meta-analysis (e.g., the study design and its conduct). Therefore, a random-effects model was used [62].

Most studies in the dataset reported multiple effect sizes from a single sample. These effect sizes ere not statistically independent because of the correlated estimation error. The robust variance estimation (RVE) approach was accordingly employed to address the data dependency [61]. This approach allowed efficient estimations of standard errors, effect sizes and confidence intervals taking into account correlations between effect sizes from the same sample. It was estimated that there would be between-study variance in the effect sizes (τ^2^) with an author-specified within-study mean correlation between effect sizes (ρ = 0.8). Sensitivity analyses indicated the results (the effect size and τ^2^) were robust across different values of ρ. As such, random-effects models with RVE correction were applied to pooled effect sizes and heterogeneity was further explored using the ROBUMETA package in R (https://cran.r-project.org/web/packages/robumeta/robumeta.pdf, accessed on 10 December 2022) [63].

### 2.4. Heterogeneity Analyses

Subgroup analyses were used to examine the mean correlation between WM and arithmetic for each subgroup of categorical moderators (i.e., WM domains, operation types, and types of arithmetic tasks). Multilevel meta-regression analyses were performed to examine the effect of each moderator on the WM-arithmetic relationship while controlling for other variables. Continuous (age) and dichotomous variables (types of arithmetic tasks) were entered directly into the models. For multiple categorical variables (WM domains and operation types), dummy coding was applied to compare each pair of subgroups [64].

### 2.5. Publication Bias

**Figure 2:**
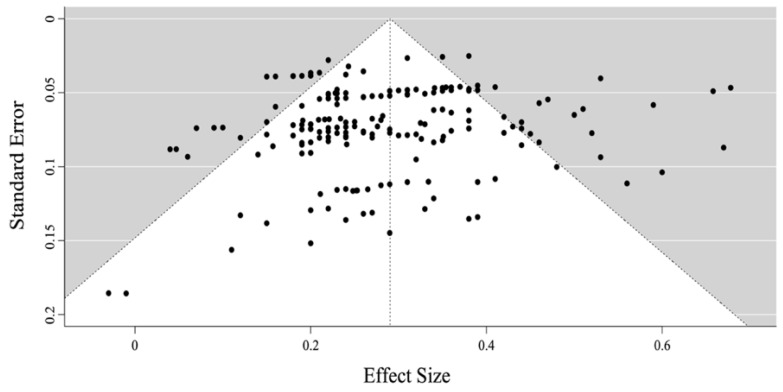
Funnel Plot of standard errors by correlation effect size.

Egger’s test and a funnel plot [65] were used to analyse publication bias, the results of which are shown in Figure 2 and discussed subsequently.

## 3. Results

### 3.1. Summary Effect Size

A frequentist approach was used for the meta-analysis for two reasons: (1) to avoid prior assumptions about the effect sizes associated with WM-arithmetic relationships given the variability of available evidence; and, (2) an interest in estimating effect sizes themselves and their associated reliability, rather than their relative probability. Meta-analysis of 46 studies with 187 *r* statistics obtained from 11224 participants (55 independent samples) revealed an overall significant and moderate correlation between WM and arithmetic, *r* = 0.312, *p* < 0.001, 95% confidence interval (CI) [0.276, 0.348].

### 3.2. The Moderation Effects of WM Domains

Verbal, visuospatial, and composite (tasks that include both verbal and visuospatial components) WM domains were included in subgroup analyses. There was a moderate correlation between arithmetic and verbal WM, *r* = 0.332, *p* < 0.001, 95% CI [0.286, 0.379], a weak correlation between arithmetic and visuospatial WM, *r* = 0.297, *p* < 0.001, 95% CI [0.243, 0.350], and a moderate correlation between arithmetic and composite WM, *r* = 0.373, *p* = 0.040, 95% CI [0.216, 0.529]. The composite WM was not included in subsequent meta-regression analyses due to the insufficient number of effect sizes (7 correlations; [66]. The mean correlations between WM and arithmetic for each category of moderation analysis can be found in Table 2.

A meta-regression model on the correlations between WM and arithmetic yielded a significant difference between verbal WM and visuospatial WM regarding the WM-arithmetic correlation, β = −0.056, *t* = −2.111, *p* = 0.041, τ^2^ = 0.011, after controlling for age, operation types, and types of arithmetic tasks (see Table 3). This indicated that verbal WM held a stronger association with arithmetic when compared with visuospatial WM.

Visuospatial WM was further sub-classified into three domains: visual, spatial-sequential, and spatial-simultaneous WM. Visual WM was excluded from the analysis since there were no specialized tasks evaluating it in the dataset. Both spatial-sequential WM, *r* = 0.316, *p* < 0.001, 95% CI [0.204, 0.428], and spatial-simultaneous WM, *r* = 0.257, *p* < 0.001, 95% CI [0.231, 0.283], yield significant correlations with arithmetic. There was no significant difference between the correlations with arithmetic for these two spatial WM domains, β = −0.159, *t* = −1.860, *p* = 0.083, τ^2^ = 0.015.

### 3.3. The Moderation Effects of Age

After controlling for WM domains, operation types, and types of arithmetic tasks, there was no significant effect of age on the correlation between WM and arithmetic, β = −0.001, *t* = −1.639, *p* = 0.109, τ^2^ = 0.011. The strength of the correlation between WM and arithmetic remained stable across ages.

To examine the effects of age on specific WM domains, four regression models were conducted on the correlations between arithmetic and (1) verbal WM, (2) visuospatial WM, (3) spatial-sequential WM, and (4) spatial-simultaneous WM. After controlling for operation types and types of arithmetic tasks, the correlation between arithmetic and verbal WM showed a significant decline with age, β = −0.003, *t* = −2.405, *p* = 0.023, τ^2^ = 0.013. That is, the correlation between verbal WM and arithmetic was stronger in younger children than in older children. In contrast, the effect of age on visuospatial WM was not significant, β = −0.001, *t* = −0.812, *p* = 0.424, τ^2^ = 0.013. The correlation between visuospatial WM and arithmetic remained stable across ages.

The effect of age was also non-significant for the correlation between spatial-sequential WM and arithmetic, β = −0.005, *t* = −1.760, *p* = 0.107, τ^2^ = 0.025, and between spatial-simultaneous WM and arithmetic, β = 0.001, *t* = 0.429, *p* = 0.690, τ^2^ = 0.000. That is, the correlations between both spatial-sequential and spatial-simultaneous WM and arithmetic remained consistent over age.

### 3.4. The Moderation Effects of Operation Types

The four types of operations separately showed significant correlations with WM: addition, *r* = 0.208, *p* = 0.007, 95% CI [0.121, 0.295], subtraction, *r* = 0.210, *p* = 0.019, 95% CI [0.165, 0.255], multiplication, *r* = 0.204, *p* = 0.013, 95% CI [0.136, 0.273], division, *r* = 0.240, *p* = 0.025, 95% CI [0.185, 0.295]. However, the number of effects for each type of operation was limited (7 to 13 effects). These estimations may therefore lack robustness.

Given that arithmetic performance was mostly indexed by a combination of scores from two or more types of operations in the present dataset, addition and subtraction were combined, as were multiplication and division. These combinations are referred to as the additive domain and the multiplicative domain, respectively. Subgroup analysis revealed that WM correlated significantly with both domains: addition and subtraction, *r* = 0.289, *p* < 0.001, 95% CI [0.244, 0.333], as well as multiplication and division, *r* = 0.212, *p* < 0.001, 95% CI [0.168, 0.256]. After controlling for age, WM domains and types of arithmetic tasks, the regression model did not show a significant difference in the correlation with WM between additive and multiplicative domains, β = −0.055, *t* = −1.981, *p* = 0.061, τ^2^ = 0.008.

Further analyses were performed for each WM domain to provide a more precise profile of the correlations between WM and arithmetic. The correlations with each WM domain were significant for addition and subtraction: verbal WM, *r* = 0.299, *p* < 0.001, 95% CI [0.244, 0.354], visuospatial WM, *r* = 0.236, *p* < 0.001, 95% CI [0.169, 0.304], and for multiplication and subtraction: verbal WM, *r* = 0.227, *p* = 0.016, 95% CI [0.208, 0.246], visuospatial WM, *r* = 0.207, *p* < 0.001, 95% CI [0.108, 0.306]. After controlling for age and types of arithmetic tasks, addition and subtraction showed a stronger correlation with verbal WM than multiplication and division, β = −0.067, *t* = −3.064, *p* = 0.007, τ^2^ = 0.007. There was no difference in correlations with visuospatial WM between the additive and multiplicative domains, β = 0.001, *t* = 0.029, *p* = 0.977, τ^2^ = 0.010.

### 3.5. The Moderation Effect of Arithmetic Task Types

The mean correlation between WM and arithmetic for each test format was significant: mental arithmetic, *r* = 0.299, *p* < 0.001, 95% CI [0.261, 0.337], written arithmetic, *r* = 0.311, *p* < 0.001, 95% CI [0.265, 0.358]. After controlling for age, WM domains, and operation types, the regression model revealed no difference between the WM—mental arithmetic correlation and the WM—written arithmetic correlation, β = −0.011, *t* = −0.422, *p* = 0.675, τ^2^ = 0.011, indicating that mental and written arithmetic are correlated with WM to a similar extent.

The correlations with each WM domain were significant for mental arithmetic: verbal WM, *r* = 0.310, *p* < 0.001, 95% CI [0.262, 0.359], visuospatial WM, *r* = 0.263, *p* < 0.001, 95% CI [0.210, 0.316], and for written arithmetic: verbal WM, *r* = 0.319, *p* < 0.001, 95% CI [0.266, 0.372], visuospatial WM, *r* = 0.322, *p* < 0.001, 95% CI [0.269, 0.376]. After controlling for age and operation types, there was no difference in correlations with verbal WM between mental and written arithmetic, β = −0.022, *t* = −0.619, *p* = 0.541, τ^2^ = 0.012, nor in correlations with visuospatial WM, β = 0.056, *t* = 1.305, *p* = 0.203, τ^2^ = 0.014. This suggests WM domains and arithmetic task are correlated to a comparable degree.

### 3.6. Publication Bias

The funnel plot (see Figure 2) showed a near symmetry in the 187 effect sizes. The Egger test indicated no significant bias (*p* = 0.074), suggesting that there was little influence of publication bias in the current meta-analysis.

## 4. Discussion

The current meta-analysis aimed to: (a) examine the relationship between WM and arithmetic in primary school children; (b) investigate whether the WM-arithmetic relationship is affected by WM domains, age, operation types, and types of arithmetic tasks. A significant and medium correlation between WM and arithmetic was found. This relationship was affected by WM domain, age, and operation types, but not by types of arithmetic tasks. These findings are discussed here.

First, regarding WM domains, both verbal and visuospatial WM were significantly correlated with arithmetic. Verbal WM was shown to have a stronger link with arithmetic than visuospatial WM. The two subtypes of visuospatial WM: spatial-sequential and spatial-simultaneous WM were correlated with arithmetic to a similar degree. Secondly, in terms of age, the overall WM-arithmetic correlation and the correlation in visuospatial WM, spatial-sequential WM, and spatial-simultaneous WM domains were not affected by age. However, the correlation between verbal WM and arithmetic was weaker in older children compared to younger children. Regarding operation types, addition, subtraction, multiplication, and division were all markedly correlated with WM. The correlation between verbal WM and arithmetic was higher in addition and subtraction than in multiplication and division. Types of arithmetic tasks (i.e., mental arithmetic and written arithmetic) showed comparable relations with WM.

### 4.1. WM Domains and Age

Consistent with existing studies [16,23], the current meta-analysis indicated that the overall WM, verbal WM, and visuospatial WM were all closely linked to arithmetic competencies in primary school children. Verbal WM showed a stronger correlation with arithmetic than visuospatial WM, which supports the notion in previous research that both verbal and visual WM are crucial factors for arithmetic competencies of primary school children, but verbal WM plays a more significant role [67,68]. This is possibly due to the additional role of verbal WM in mathematical facts retrieval [19]. When performing arithmetic tasks, children are primarily involved in two cognitive processes, which are carrying out calculation steps and retrieving knowledge from long-term memory. For carrying out calculation steps, children represent and manipulate numbers in the mental model, which largely requires visuospatial WM [69]. In this process, children also need to keep relevant information (e.g., the operands and interim values) activated in their minds through verbal rehearsal, which relies on verbal WM [19,68,70]. In addition, verbal WM is implicated in the retrieval of mathematical facts from long-term memory, and the association of them to information used during the calculation [71,72]. Therefore, when performing calculations, primary school children may predominantly rely on verbal WM that is supplemented by visuospatial resources [2].

However, a meta-analysis by Peng et al. [73] found that verbal WM and visuospatial WM correlated with arithmetic to a similar degree, indicating no moderation effects of WM domains. Given that Peng, Namkung, Barnes and Sun [73] collected the data throughout the life span, whereas the present study focused on a specific age range during primary school, it is possible that despite the similar contribution of each domain of WM to arithmetic across the life span, verbal WM holds a greater impact on arithmetic than visuospatial WM during primary school [74].

No effect of age on the correlation between the overall WM and arithmetic was found. This pattern is well-observed in previous studies, in that the correlation between WM and arithmetic remains stable from mid-childhood (about 8 years old) to adulthood [20,23].

However, the comparison of verbal and visuospatial WM showed distinct developmental patterns in relation to arithmetic. There was a significant decline in the correlation between verbal WM and arithmetic throughout primary school, but the correlation between visuospatial WM and arithmetic remained stable. The reduction in the correlation between verbal WM and arithmetic partly supports evidence of an age-related shift from verbal WM to visuospatial WM [25]. The current results support findings from previous research [69], that verbal WM is important for arithmetic in lower grades but the predictive power wanes as the grade level progresses. Furthermore, verbal WM has been linked to simple addition and multiplication problems to maintain the operands and interim results [15] but possibly plays a limited role in complex operations, such as those required in older children, where the answers cannot be retrieved directly from memory [75]. As operations become increasingly complex in upper grades, the strong verbal representation of arithmetic problems may be transformed into representations and manipulations of numbers and abstract symbols that are visuo-spatially based [26]. Such claims were supported by neuroimaging studies that found brain activation patterns during calculation tasks showed a shift from the frontal area (which is mostly involved in verbal WM) to the parietal areas (which is involved in visuospatial WM) [76,77]. The current study thus indicated a downward trend in the correlation between verbal WM and arithmetic during primary school.

The shift from verbal to visuospatial WM, however, was not fully supported by the present results. A relatively stable correlation between visuospatial WM and arithmetic that did not vary with age was found. The long-lasting importance of visuospatial WM on arithmetic during primary school fits well with the evidence from Allen, Higgins and Adams [30] and Vieira, Ribeiro, Farias and Freitas [16]. It appears reasonable that using visual and spatial representations to manipulate numbers using mental images may serve as an effective approach for calculation in both younger and elder children [78]. This is in line with descriptions of visuospatial WM that posit this ability serves as a mental blackboard supporting representations and manipulations of numbers [69,79].

Consistent with a previous meta-analysis [30], spatial-sequential and spatial-simultaneous WM were equally correlated with arithmetic. When doing calculations, it might be important to hold information sequentially when coding at different time points such as carrying and combining intermedium values [80], which is supposed to rely on spatial-sequential WM. However, the correlation between spatial-simultaneous WM and arithmetic is less understood. Allen et al. (2020) argued that in both spatial-simultaneous WM tasks and most arithmetic tasks, information is displayed simultaneously and available since then, thus the correlation may be a by-product of how information is presented. This claim needs further scrutiny by considering the way that information is presented in arithmetic tasks.

### 4.2. Operation Types

Consistent with previous evidence [19,20], both verbal and visuospatial WM were significantly correlated with operation type (i.e., addition, subtraction, multiplication, and division). Given there were only arithmetic tasks for every single type of operation in very few studies, addition and subtraction were combined to form an additive domain; and multiplication was combined with division to form a multiplicative domain. The results showed that verbal WM correlated with the additive domain to a greater degree than with the multiplicative domain, whereas visuospatial WM correlated with both additive and multiplicative domains to a similar degree. The importance of verbal WM in the additive domain fits well with evidence from Rasmussen and Bisanz [27] which found verbal WM to be a better predictor of addition calculation performance compared to other WM domains. Similarly, an analysis by Fuchs et al. [81] showed a significant link for verbal WM and phonological processing, with addition and subtraction in grade-3 children. The present study, together with the evidence discussed here, indicates a predominant role of verbal WM in arithmetic, especially in addition and subtraction.

However, the findings discussed here contradict evidence that has found no difference in contributions of WM to addition, subtraction, multiplication, and division in children aged 7–12 [20]. This is further complicated when considering findings by van der Ven, van der Maas, Straatemeier and Jansen [19], which revealed a stronger correlation of visuospatial WM with addition and subtraction than with multiplication and division. However, studies addressing the relationship between WM and four operations simultaneously remain remarkably scarce, and the differentiation of cognitive processes among addition, subtraction, multiplication, and division are not clear yet. Thus, the present study can be regarded as contributing to subsequent investigations into the WM-arithmetic relationship when considering operation type.

### 4.3. Types of Arithmetic Tasks

Although mental arithmetic is posited to rely on WM to a greater extent than written arithmetic [2,46], the current results found no difference between mental and written arithmetic with regard to the correlation with WM. This is supported by evidence from Allen and Dowker [48] who found that visuo-spatial WM was important in spoken and mental written arithmetic. However, it should be noted that the task difficulty varies between mental and written arithmetic tasks in existing studies, which potentially affects the involvement of WM in these tasks [82]. As noted by Robert and LeFevre [83], calculations with large operands require more involvement of WM than calculations with small operands. Likewise, single-step calculations rely less on WM than multi-step calculations [84]. Given tasks with small operands (e.g., the computational fluency test) are used as measures of mental arithmetic ability in most studies, and multi-step operations with small-to-large operands are mostly used as measures of written arithmetic ability, it may be the case that mental arithmetic tasks used have required less reliance on WM due to a lower level of difficulty. This might offset the increasing WM demands due to the greater memory load when children cannot take notes on paper in mental arithmetic tasks.

### 4.4. Limitations and Implications

The current meta-analysis used two datasets (i.e., ERIC and PsycInfo), to examine the relationship between WM and arithmetic in primary children. These two databases provide considerable access to the two relevant fields of education and psychology. However, it is noted that the use of these two databases alone might have led to a degree of bias in favour of studies conducted in North America. In addition, the use of databases for other disciplines (e.g., neuroimaging) might increase the number of studies reporting correlations between WM and types of arithmetic operations. This would allow for a fuller examining of the effect of operation type, such as those suggested by findings from other researchers [85].

There are few studies with primary school children which investigate the correlation between visual WM and arithmetic. Given that visual WM was has been related to mathematical performance separate from spatial WM in some studies [33], there is value in examining the role of visual WM in arithmetic. Again, this might be achieved by broadening the literature search to other databases.

It is acknowledged that the age limit in the current study may not be broad enough to detect the significant changes in the role of visuospatial WM in arithmetic as children develop. Existing evidence from adolescents and adult studies has suggested a crucial role of visuospatial WM over and above verbal WM in arithmetic [28,86]. There is possibly an increasing role of visuospatial WM in arithmetic from late childhood to adulthood. It is recommended that future meta-analyses include an older age group to potentially identify this relationship.

Despite the compelling evidence supporting the correlation between WM and arithmetic in the present analysis, some alternative designs may also provide useful information on the predictive role of WM in the development of arithmetic ability. One approach to understanding causal links between WM and arithmetic is a meta-analysis of interventional designs, which investigate the gains in both WM and arithmetic skills after training of WM [87]. A second approach is examining dual-task experiments, in which children are required to perform a primary task (e.g., arithmetic) and simultaneously complete a secondary task (e.g., WM tasks). The comparison of accuracy and reaction time between a single-task condition and dual-task conditions may provide sensitive detection of differential contributions of specific WM domains to arithmetic [1].

## 5. Conclusions

The present study provided insights into the relationship between WM and arithmetic skills in primary school children: First, WM domain influenced the correlations between WM and arithmetic, in that verbal WM correlated with arithmetic to a greater extent than visuospatial WM. Second, the correlation between verbal WM and arithmetic declined with age, whereas the influence of visuospatial WM, spatial-sequential WM, and spatial-simultaneous WM on arithmetic remained stable during primary school. Third, the only effect on operation types was from verbal WM, whereby the verbal WM-additive correlation was stronger than the verbal WM-multiplicative correlation. Lastly, mental and written arithmetic correlated with WM to a similar degree. Such findings provide a more in depth understanding of where and how WM plays an important role in arithmetic abilities. These findings can inform reasonable adjustments and intervention studies for children who might struggle with arithmetic due to deficits in WM.

## Figures and Tables

**Table 1:** Descriptions of WM domains, subtypes of spatial WM, and arithmetic task types.

	Task Descriptions	Examples
**WM Domains**		
Verbal WM	Tasks that involve processing and maintaining verbal information. Verbal information includes both letters and digits.	Backward digit span, backward letter span, counting span, listening span, WJ-III Numbers Reversed [50], WMTB-C Listening Recall [51], sentence span
Visuospatial WM	Tasks that involve processing and maintaining visual (i.e., object features such as shape, size, and colour) and/or spatial information (i.e., locations).	Forward or backward block span, Corsi block, visual matrix task, spatial-sequential span, spatial-simultaneous span, odd-one-out task, mapping and directions task, Mr. X task, maze memory, eye chart E task
Composite WM	Tasks that involve processing and maintaining the information within more than one domain: verbal, visual, and spatial, or composite scores of WM tasks across domains.	Composite of odd-one-out, spatial recall, listening recall, backward word span, composite of colour span backward and digit span backward and listening span, composite of Corsi block and forward digit span and backward digit span
**Subtypes of Spatial WM**		
Spatial-sequential WM	Tasks that require processing and recalling a sequence of spatial locations in a specific order.	Corsi block, the block forward span, the block backward span, automated symmetry span, spatial span, block recall, spatial-sequential task
Spatial-simultaneous WM	Tasks that require recalling an array of spatial locations that have been presented simultaneously.	S-CPT visual matrix task [52], visual matrix span, spatial-simultaneous task
**Arithmetic Task Types**		
Mental arithmetic	Arithmetical calculations performed mentally, with no help from any external apparatus or or devices such as writting figures down, usually with a short-time limit (e.g., 1 min for 25 calculations).	Fact Fluency subtests of the Grade 3 Math Battery [53], Test of Computational Fluency [54], Math fact fluency, Arithmetic fact retrieval, 2-grade calculations battery-arithmetic [55], WISC mental arithmetic [56]
Written arithmetic	Arithmetical calculations performed with the help of external apparatus or devices such as pencil and paper.	Double-Digit Addition and Subtraction subtests of the Grade 3 Math Battery [53], WRAT-III arithmetic [57], WIAT numerical operations [58], SDMT-4 computation [59], KeyMath A arithmetic, The heidelberg mathematics test [60], BAS Arithmetic

Note: WJ-III Numbers Reversed = the Woodcock-Johnson Psycho-Educational Battery Revised subtests of concept formation; WMTB-C Listening Recall = the Woodcock Memory Test Battery—Children listening recall subtest; S-CPT visual matrix task = Swanson Cognitive Processing Test Visual Matrix subtest; WISC mental arithmetic = Wechsler Intelligence Scale for Children—mental arithmetic; WRAT-III arithmetic = The third edition of the Wide Range Achievement Test—arithmetic subset; WIAT numerical operations = the numerical operations subtest for the Wechsler Individual Achievement Test; SDMT—4 computation = The Computation subtest of the Stanford Diagnostic Math Test–Fourth Edition; BAS Arithmetic = British Abilities Scales Basic Number Skills subset.

**Table 2:** The mean correlation between working memory and arithmetic for each category.

The Subgroup of Categorical Moderators	*n*	*r*	95% CI	*p*	τ^2^
**Domains of WM**					
Verbal WM	93	0.332 ***	[0.286, 0.379]	0.000001	0.014
Visuospatial WM	87	0.297 ***	[0.243, 0.350]	0.000	0.013
Composite WM	7	0.373 *	[0.216, 0.529]	0.040	0.033
Spatial-sequential WM	36	0.316 ***	[0.204, 0.428]	0.000	0.030
Spatial-simultaneous WM	20	0.257 ***	[0.231, 0.283]	0.000	0.000
**Operation Types**					
Addition	12	0.208 **	[0.121, 0.295]	0.007	0.011
Subtraction	7	0.210 *	[0.165, 0.255]	0.019	0.000
Multiplication	13	0.204 *	[0.136, 0.273]	0.013	0.000
Division	10	0.240 *	[0.185, 0.295]	0.025	0.000
Additive domain	63	0.289 ***	[0.244, 0.333]	0.000	0.009
Multiplicative domain	23	0.212 ***	[0.168, 0.256]	0.000	0.000
**Types of Arithmetic Tasks**					
Mental Arithmetic	86	0.299 ***	[0.261, 0.337]	0.000	0.008
Written Arithmetic	98	0.311 ***	[0.265, 0.358]	0.000	0.010

Note: *n* = the number of effect sizes. Additive = addition and subtraction. Multiplicative = multiplication and division. * *p* < 0.05. ** *p* < 0.01. *** *p* < 0.001.

**Table 3:** Meta-regression on the correlation between working memory and arithmetic.

Variables	β	*SE*	*t*	95% CI	*p*
**Domains of WM**					
Verbal vs. visuospatial	−0.056	0.026	−2.111 *	[−0.108, −0.004]	0.041
Spatial-sequential vs. spatial-simultaneous	−0.159	0.086	−1.860	[−0.326, 0.012]	0.083
**Age**					
Age (the overall WM)	−0.001	0.001	−1.639	[−0.003, 0.000]	0.109
Age (verbal WM)	−0.003	0.001	−2.405 *	[−0.005, 0.000]	0.023
Age (visuospatial WM)	−0.001	0.001	−0.812	[−0.004, 0.001]	0.424
Age (spatial-sequential WM)	−0.005	0.003	−1.760	[−0.007, 0.004]	0.107
Age (spatial-simultaneous WM)	0.001	0.003	0.429	[−0.005, 0.007]	0.690
**Operation Types**					
Additive vs. multiplicative (the overall WM)	−0.055	0.028	−1.981	[−0.110, −0.001]	0.061
Additive vs. multiplicative (verbal WM)	−0.067	0.022	−3.064 **	[−0.110, −0.024]	0.007
Additive vs. multiplicative (visuospatial WM)	0.001	0.048	0.029	[−0.092, 0.095]	0.977
**Types of Arithmetic Tasks**					
Mental vs. written (the overall WM)	−0.011	0.027	−0.422	[−0.063, 0.041]	0.675
Mental vs. written (verbal WM)	−0.022	0.036	−0.619	[−0.081, 0.058]	0.541
Mental vs. written (visuospatial WM)	0.056	0.043	1.305	[−0.096, 0.074]	0.203

Note: The first group in each group comparison variable is the reference group (e.g., in verbal vs. visuospatial, verbal is the reference group in the dummy coding). Additive = addition and subtraction. Multiplicative = multiplication and division. * *p* < 0.05. ** *p* < 0.01.

## Data Availability

Not applicable.

## Supplementary Materials

The following are available online at https://www.mdpi.com/article/10.3390/brainsci13010022/s1, File S1: Key characteristics of included studies of the meta-analysis. File S2: Reference list of all studies included in meta-analysis. References [88,89,90,91,92,93,94,95,96,97,98,99,100,101,102,103,104,105,106,107,108,109,110,111,112,113,114,115,116,117,118,119,120,121,122,123,124,125,126,127] are cited in the supplementary materials.
