# Supplementary material for: The Relationship between Working Memory and Arithmetic in Primary School Children: A Meta-Analysis
Source: Brain Sci. 2022 Dec 22;13(1):22. doi: 10.3390/brainsci13010022 (PMC9856839; doi:10.3390/brainsci13010022)
Supplement: Supplementary file 1 [file brainsci-13-00022-s001.zip › File S1.pdf]

File S1

File S1. Key characteristics of included studies of the meta-analysis

study	n	Age	r	WM measures	arithmetic measures
Seethaler et al., 2006	315	104	0.220	WJ-III Numbers Reversed	Addition and Subtraction Fact Fluency Test
	315	104	0.270	WJ-III Numbers Reversed	Double-Digit Addition and Subtraction subtests
	315	104	0.340	WMTB-C Listening Recall	Addition and Subtraction Fact Fluency Test
	315	104	0.310	WMTB-C Listening Recall	Double-Digit Addition and Subtraction subtests
Swanson, 2006	319	87.12	0.530	Listening span, Backward digit span, Sentence task, Semantic association task	WRAT—III, WIAT, Test of Computational Fluency
	319	87.12	0.380	Visual-Matrix task, Mapping and direction task	WRAT—III, WIAT, Test of Computational Fluency
Lundberg et al., 2006	60	118	0.480	Backward digit span (WISC)	Mental calculation
	60	118	0.530	Backward digit span (WISC)	Nonverbal algorithm compotation
	60	106	0.390	Backward digit span (WISC)	Mental calculation
	60	106	0.410	Backward digit span (WISC)	Nonverbal algorithm compotation
Fuchs et al., 2006	312	104.76	0.260	WMTB-C Listening Recall	Math Fact Fluency test
	312	104.76	0.280	WMTB-C Listening Recall	Double-Digit Addition and Subtraction subtests
	312	104.76	0.220	WJ-III Numbers Reversed	Math Fact Fluency test
	312	104.76	0.210	WJ-III Numbers Reversed	Double-Digit Addition and Subtraction subtests
Holmes et al., 2006	148	106.93	0.260	The maze memory task	Mental arithmetic developed from Qualifications and Curriculum Authority
	148	106.93	0.510	Listening recall task	Mental arithmetic

					developed from Qualifications and Curriculum Authority
Swanson et al., 2007	353	87	0.360	Backward digit span	WRAT- III
	353	87	0.350	Backward digit span	WIAT
	353	87	0.290	Backward digit span	Math Fact Fluency test
	353	87	0.370	Sentence span	WRAT- III
	353	87	0.390	Sentence span	WIAT
	353	87	0.230	Sentence span	Math Fact Fluency test
	353	87	0.370	Digit-sentence span	WRAT- III
	353	87	0.350	Digit-sentence span	WIAT
	353	87	0.310	Digit-sentence span	Math Fact Fluency test
	353	87	0.290	Visual matrix task	WRAT- III
	353	87	0.300	Visual matrix task	WIAT
	353	87	0.240	Visual matrix task	Math Fact Fluency test
	353	87	0.320	Mapping and directions	WRAT- III
	353	87	0.310	Mapping and directions	WIAT
	353	87	0.220	Mapping and directions	Math Fact Fluency test
Holmes et al., 2008	51	91	0.150	The visual patterns test	Mental arithmetic developed from Qualifications and Curriculum Authority
	51	91	0.260	The block recall task	Mental arithmetic developed from Qualifications and Curriculum Authority
	51	91	0.270	The visual patterns test, the block recall task	Mental arithmetic developed from Qualifications and Curriculum Authority
	56	115	0.220	The visual patterns test	Mental arithmetic developed from Qualifications and Curriculum Authority
	56	115	0.120	The block recall task	Mental arithmetic developed from Qualifications and Curriculum Authority
	56	115	0.200	The visual patterns test, the block recall task	Mental arithmetic developed from Qualifications and Curriculum Authority

Berg, 2008	90	121.5	0.520	Semantic categorization	WRAT- III
	90	121.5	0.320	Auditory digit sequencing	WRAT- III
	90	121.5	0.440	Visual matrix task	WRAT- III
	90	121.5	0.460	Corsi block	WRAT- III
Fuchs et al., 2010	205	73.44	0.470	WMTB-C Listening recall	Math Fact Fluency test
	205	73.44	0.350	Block recall, Maze memory	Math Fact Fluency test
Lee et al., 2011	151	120.58	0.290	Listening recall task	WIAT (group 1)
	151	120.58	0.240	Listening recall task	WIAT (group 2)
	151	120.58	0.230	Listening recall task	WIAT (group 3)
	151	120.58	0.260	Mr. X task	WIAT (group 1)
	151	120.58	0.120	Mr. X task	WIAT (group 2)
	151	120.58	0.180	Mr. X task	WIAT (group 3)
	142	132.58	0.240	Listening recall task	WIAT (group 1)
	142	132.58	0.220	Listening recall task	WIAT (group 2)
	142	132.58	0.210	Listening recall task	WIAT (group 3)
	142	132.58	0.290	Mr. X task	WIAT (group 1)
	142	132.58	0.220	Mr. X task	WIAT (group 2)
	142	132.58	0.270	Mr. X task	WIAT (group 3)
Lan et al., 2011	119	60.24	0.440	the Sentence completion task	verbal calculation
	139	58.8	0.240	the Sentence completion task	verbal calculation
Seethaler et al., 2011	688	96	0.200	Listening recall task	Math Fact Fluency test
	688	96	0.210	WJ-III Numbers Reversed	Math Fact Fluency test
	688	96	0.260	Listening recall task	WRAT- III
	688	96	0.260	WJ-III Numbers Reversed	WRAT- III
Zheng et al., 2011	310	105.36	0.390	Listening sentence span	WIAT
	310	105.36	0.380	Listening sentence span	WRAT- III
	310	105.36	0.310	Digit-sentence span	WIAT
	310	105.36	0.330	Digit-sentence span	WRAT- III
	310	105.36	0.260	Visual matrix task	WIAT
	310	105.36	0.290	Visual matrix task	WRAT- III
	310	105.36	0.230	Mapping and directions	WIAT
	310	105.36	0.240	Mapping and directions	WRAT- III
Simmons et al., 2012	41	70	0.110	Mazes memory test, Block recall test	Mental addition

	41	70	0.670	Odd-one-out, spatial recall, listening recall, backward word span	Mental addition
	49	95	0.330	Mazes memory test, Block recall test	Mental multiplication
	49	95	0.240	Odd-one-out, spatial recall, listening recall, backward word span	Mental multiplication
	66	76.5	0.252	Corsi blocks, Matrics task	Addition
	66	76.5	0.253	Corsi blocks, Matrics task	Multiplication
	66	76.5	0.334	Corsi blocks, Matrics task	Division
Arndt et al., 2013	66	76.5	0.265	Colour span backward, Digit span backward, Listening span	Addition
	66	76.5	0.248	Colour span backward, Digit span backward, Listening span	Multiplication
	66	76.5	0.211	Colour span backward, Digit span backward, Listening span	Division
	357	105.97	0.228	WMTB-C counting recall	WIAT addition fluency test
Jordan et al., 2013	357	105.97	0.354	WMTB-C counting recall	WRAT-4
	357	105.97	0.341	WMTB-C counting recall	WRAT-4
Vukovic et al., 2013	113	94	0.200	S-CPT Visual matrix task	SDMT-4 computation
	113	94	0.190	S-CPT Visual matrix task	SDMT-4 computation
Träff, 2013	134	142	0.300	Listening span	Arithmetic fact retrieval
	134	142	0.270	Listening span	Arithmetic calculation task
	134	142	0.380	Visual matrix span	Arithmetic fact retrieval
	134	142	0.380	Visual matrix span	Arithmetic calculation task
Vukovic et al., 2013	167	82	0.240	S-CPT Visual matrix task	SDMT-4 computation
	167	82	0.220	S-CPT Visual matrix	SDMT-4 computation

				task	
	167	82	0.250	S-CPT Visual matrix task	SDMT-4 computation
	167	82	0.190	S-CPT Visual matrix task	SDMT-4 computation
	167	94	0.230	S-CPT Visual matrix task	SDMT-4 computation
	167	94	0.200	S-CPT Visual matrix task	SDMT-4 computation
	167	94	0.200	S-CPT Visual matrix task	SDMT-4 computation
LeFevre et al., 2013	157	118	0.210	Backward digit span	Key Math A/WJ-R arithmetic
	157	118	0.330	Spatial span	Key Math A/WJ-R arithmetic
	157	118	0.220	Backward digit span	Key Math A/WJ-R arithmetic
	157	118	0.150	Spatial span	Key Math A/WJ-R arithmetic
Kleemans et al., 2014	129	99.34	0.047	Number recall	Addition
	129	99.34	0.190	Number recall	Subtraction
	129	99.34	0.040	Memory span	Addition
	129	99.34	0.157	Memory span	Subtraction
Martin et al., 2014	189	73.92	0.360	Shape and position recall	Math Fact Fluency test
	189	73.92	0.270	Shape and position recall	WRAT- III
	192	73.92	0.460	Digit backward span	Math Fact Fluency test
	192	73.92	0.380	Digit backward span	WRAT- III
Fuchs et al., 2015	206	90	0.340	WMTB-C Counting recall	Arithmetic combinations
	206	90	0.350	WMTB-C Listening recall	Arithmetic combinations
Mix et al., 2016	854	107.81	0.243	Visual matrix task	Whole number calculation
Cirino et al., 2016	162	148.8	0.276	Automated Symmetry Span	Single-digit addition and multiplication
	162	148.8	0.325	Automated Symmetry Span	Procedural computation
Skagerlund et al., 2016	133	116.28	0.320	Visual matrix task	Multidigit calculation
	133	116.28	0.360	Visual matrix task	Arithmetic equations
	133	116.28	0.310	Visual matrix task	Arithmetic fact retrieval
	133	116.28	0.220	Sentence span	Multidigit calculation
	133	116.28	0.200	Sentence span	Arithmetic equations

	133	116.28	0.190	Sentence span	Arithmetic fact retrieval
Swanson & Fung, 2016	392	100.68	0.230	The mapping and direction span, the visual matrix	WRAT- III, WIAT
Liu et al., 2017	30	133.08	0.130	digit span backward	multiple-step 3-digit multiplication
	30	133.08	0.100	digit span backward	single-step 3-digit multiplication
	41	133.08	0.390	phonological loop task	multiple-step 2-digit multiplication
	41	133.08	0.290	phonological loop task	multiple-step 2-digit multiplication
	41	133.08	0.380	eye chart E task	multiple-step 2-digit multiplication
	41	133.08	0.200	eye chart E task	multiple-step 2-digit multiplication
Wong, 2017	124	73	0.241	Backward digit span	Arithmetic computation
Zhang et al., 2017	346	95	0.220	Backward digit span	Written computation
Ching et al., 2017	115	76.32	0.350	Digit span backward, counting recall	Addition and Subtraction
	115	76.32	0.420	Digit span backward, counting recall	Addition and Subtraction
	115	76.32	0.060	Corsi block	Addition and Subtraction
	115	76.32	0.140	Corsi block	Addition and Subtraction
Korhonen et al., 2018	624	112.2	0.190	Operation span	Written arithmetic
	624	112.2	0.240	Operation span	Numeric understanding and mental arithmetic
	624	112.2	0.200	Digit backward span	Written arithmetic
	624	112.2	0.200	Digit backward span	Numeric understanding and mental arithmetic
	624	112.2	0.180	The block forward span	Written arithmetic
	624	112.2	0.160	The block forward span	Numeric understanding and mental arithmetic
	624	112.2	0.150	The block backward span	Written arithmetic
	624	112.2	0.180	The block backward span	Numeric understanding and mental arithmetic
Träff et al., 2018	269	114.72	0.190	Complex word repetition	Arithmetic fluency test
	269	114.72	0.230	Complex word repetition	Arithmetic fact retrieval

	269	114.72	0.160	Complex word repetition	Multi-digit calculation
Fuchs et al., 2018	325	90	0.360	WMTB-C Listening recall	2-grade calculations battery-arithmetic
	325	90	0.350	WMTB-C Listening recall	Addition and subtraction
	325	90	0.380	WMTB-C Counting recall	2-grade calculations battery-arithmetic
	325	90	0.410	WMTB-C Counting recall	Addition and subtraction
Morris et al., 2019	135	91.97	0.678	Backward spatial span	WIAT numerical operations
	135	91.97	0.658	Backward digit span	WIAT numerical operations
Georges at al., 2019	68	114.36	0.240	Visual matrix task	The Heidelberg mathematics test-addition
	68	114.36	0.290	Visual matrix task	The Heidelberg mathematics test-subtraction
	68	114.36	0.230	Visual matrix task	The Heidelberg mathematics test-multiplication
	68	114.36	0.280	Visual matrix task	The Heidelberg mathematics test-division
	68	114.36	0.310	Visual matrix task	The Heidelberg mathematics test-addition and subtraction
Soltani et al., 2020	113	94.92	0.340	Counting span	Mental arithmetic
Yang et al., 2020	155	93	0.420	Backward digit span	addition, subtraction, and multiplication
	155	93	0.380	Backward digit span	addition, subtraction, and multiplication
Allen et al., 2020	197	95.99	0.221	Spatial-simultaneous 4×3 matrix	Access Mathematics Test calculating
	197	95.99	0.282	Spatial-simultaneous 4×4 matrix	Access Mathematics Test calculating
	197	95.99	0.191	Spatial-sequential 3×3 matrix (no order)	Access Mathematics Test calculating
	197	95.99	0.209	Spatial-sequential 4×4 matrix (no order)	Access Mathematics Test calculating

	197	95.99	0.150	Block recall	Access Mathematics Test calculating
	197	95.99	0.234	WMTB-C Backward digit span	Access Mathematics Test calculating
	197	95.99	0.216	WMTB-C Counting recall	Access Mathematics Test calculating
Magalhaes et al., 2021	134	121.56	0.440	Listening sentence span	Numerical operations
	134	121.56	0.500	Listening sentence span	Arithmetic fluency test
Xu et al., 2021	182	93.6	0.190	Digit backward span	Addition
	182	93.6	0.280	Digit backward span	Subtraction
	182	93.6	0.180	Spatial span	Addition
	182	93.6	0.200	Spatial span	Subtraction
	182	93.6	0.200	Digit backward span	Addition
	182	93.6	0.250	Digit backward span	Subtraction
	182	93.6	0.240	Digit backward span	Multiplication
	182	93.6	0.100	Spatial span	Addition
	182	93.6	0.090	Spatial span	Subtraction
	182	93.6	0.070	Spatial span	Multiplication
Rivella et al., 2021	122	115.56	0.326	Forward span/backward span/dot-matrix	the AC-FL calculation
	122	115.56	0.351	forward word span/backward word span/listening span	the AC-FL calculation
Scofield et al., 2021	1157	152.63	0.220	Digit backward span	Arithmetic fluency test
	1157	152.63	0.380	Digit backward span	Fractions arithmetic
	1157	152.63	0.310	Corsi block tapping	Arithmetic fluency test
	1157	152.63	0.350	Corsi block tapping	Fractions arithmetic
Xu et al., 2021	106	97	0.450	digit forward span, digit backward span, spatial span	Arithmetic fluency test
	54	98	0.340	digit forward span, digit backward span, spatial span	Arithmetic fluency test
Vieira et al., 2021	126	99.96	0.590	Corsi block tapping	School achievement test-arithmetic
	126	99.96	0.430	WISC digit span	School achievement test-arithmetic
Allen et al., 2022	39	89.45	0.600	Dot matrix	BAS arithmetic
	39	89.45	0.560	Dot matrix	WISC-mental arithmetic
