# Supplementary material for: The Relationship between Working Memory and Arithmetic in Primary School Children: A Meta-Analysis
Source: Brain Sci. 2022 Dec 22;13(1):22. doi: 10.3390/brainsci13010022 (PMC9856839; doi:10.3390/brainsci13010022)
Supplement: Supplementary file 1 [file brainsci-13-00022-s001.zip › File S2.pdf]

File S2

List of all studies included in meta-analysis

1. Allen, K.; Giofrè, D.; Higgins, S.; Adams, J. Working memory predictors of written mathematics in 7-to 8-year-old children. Quarterly Journal of Experimental Psychology 2020, 73, 239-248.
2. Allen, L.; Dowker, A. Spatial Working Memory Counts: Evidence for a Specific Association Between Visuo-spatial Working Memory and Arithmetic in Children. International Electronic Journal of Elementary Education 2022, 14, 199-211.
3. Arndt, D.; Sahr, K.; Opfermann, M.; Leutner, D.; Fritz, A. Core knowledge and working memory as prerequisites of early school arithmetic. South African Journal of Childhood Education 2013, 3, 01-20.
4. Berg, D.H. Working memory and arithmetic calculation in children: The contributory roles of processing speed, short-term memory, and reading. Journal of experimental child psychology 2008, 99, 288-308.
5. Ching, B.H.-H.; Nunes, T. The importance of additive reasoning in children's mathematical achievement: A longitudinal study. Journal of Educational Psychology 2017, 109, 477.
6. Cirino, P.T.; Tolar, T.D.; Fuchs, L.S.; Huston-Warren, E. Cognitive and numerosity predictors of mathematical skills in middle school. Journal of Experimental Child Psychology 2016, 145, 95-119.
7. Fuchs, L.S.; Fuchs, D.; Compton, D.L.; Hamlett, C.L.; Wang, A.Y. Is word-problem solving a form of text comprehension? Scientific Studies of Reading 2015, 19, 204-223.
8. Fuchs, L.S.; Fuchs, D.; Compton, D.L.; Powell, S.R.; Seethaler, P.M.; Capizzi, A.M.; Schatschneider, C.; Fletcher, J.M. The cognitive correlates of third-grade skill in arithmetic, algorithmic computation, and arithmetic word problems. Journal of Educational Psychology 2006, 98, 29.
9. Fuchs, L.S.; Geary, D.C.; Compton, D.L.; Fuchs, D.; Hamlett, C.L.; Bryant, J.D. The contributions of numerosity and domain-general abilities to school readiness. Child development 2010, 81, 1520-1533.
10. Fuchs, L.S.; Gilbert, J.K.; Fuchs, D.; Seethaler, P.M.; N. Martin, B. Text comprehension and oral language as predictors of word-problem solving: Insights into word-problem solving as a form of text comprehension. Scientific Studies of Reading 2018, 22, 152-166.
11. Georges, C.; Cornu, V.; Schiltz, C. Spatial skills first: The importance of mental rotation for arithmetic skill acquisition. Journal of Numerical Cognition 2019, 5, 5-23.
12. Greiner de Magalhães, C.; Mervis, C.B.; Cardoso-Martins, C. Cognitive predictors of arithmetic, reading, and spelling in Brazilian Portuguese-speaking children. Reading and Writing 2021, 34, 171-198.
13. Holmes, J.; Adams, J.W. Working memory and children's mathematical skills: Implications for mathematical development and mathematics curricula. Educational Psychology 2006, 26, 339-366.
14. Holmes, J.; Adams, J.W.; Hamilton, C.J. The relationship between visuospatial sketchpad capacity and children's mathematical skills. European Journal of Cognitive Psychology 2008, 20, 272-289.
15. Jordan, N.C.; Hansen, N.; Fuchs, L.S.; Siegler, R.S.; Gersten, R.; Micklos, D. Developmental predictors of fraction concepts and procedures. Journal of experimental child psychology 2013, 116, 45-58.
16. Kleemans, T.; Segers, E.; Verhoeven, L. Cognitive and linguistic predictors of basic arithmetic skills: Evidence from first-language and second-language learners. International Journal of Disability, Development and Education 2014, 61, 306-316.
17. Korhonen, J.; Nyroos, M.; Jonsson, B.; Eklöf, H. Additive and multiplicative effects of working memory and test anxiety on mathematics performance in grade 3 students. Educational Psychology 2018, 38, 572-595.
18. Lan, X.; Legare, C.H.; Ponitz, C.C.; Li, S.; Morrison, F.J. Investigating the links between the subcomponents of executive function and academic achievement: A cross-cultural analysis of Chinese and American preschoolers. Journal of experimental child psychology 2011, 108, 677-692.
19. Lee, K.; Ng, S.F.; Bull, R.; Pe, M.L.; Ho, R.H.M. Are patterns important? An investigation of the relationships between proficiencies in patterns, computation, executive functioning, and algebraic word problems. Journal of Educational Psychology 2011, 103, 269.
20. LeFevre, J.-A.; Berrigan, L.; Vendetti, C.; Kamawar, D.; Bisanz, J.; Skwarchuk, S.-L.; Smith-Chant, B.L. The role of executive attention in the acquisition of mathematical skills for children in Grades 2 through 4. Journal of experimental child psychology 2013, 114, 243-261.
21. Liu, R.-D.; Ding, Y.; Xu, L.; Wang, J. Involvement of working memory in mental multiplication in Chinese elementary students. The Journal of Educational Research 2017, 110, 380-390.
22. Lundberg, I.; Sterner, G. Reading, arithmetic, and task orientation—How are they related? Annals of dyslexia 2006, 56, 361-377.

23. Martin, R.B.; Cirino, P.T.; Sharp, C.; Barnes, M. Number and counting skills in kindergarten as predictors of grade 1 mathematical skills. Learning and individual differences 2014, 34, 12-23.
24. Mix, K.S.; Levine, S.C.; Cheng, Y.-L.; Young, C.; Hambrick, D.Z.; Ping, R.; Konstantopoulos, S. Separate but correlated: The latent structure of space and mathematics across development. Journal of Experimental Psychology: General 2016, 145, 1206.
25. Morris, S.; Farran, E.K.; Dumontheil, I. Field independence associates with mathematics and science performance in 5-to 10-year-olds after accounting for domain-general factors. Mind, Brain, and Education 2019, 13, 268-278.
26. Rivella, C.; Cornoldi, C.; Caviola, S.; Giofrè, D. Learning a new geometric concept: The role of working memory and of domain-specific abilities. British Journal of Educational Psychology 2021, 91, 1537-1554.
27. Scofield, J.E.; Hoard, M.K.; Nugent, L.; LaMendola V, J.; Geary, D.C. Mathematics Clusters Reveal Strengths and Weaknesses in Adolescents' Mathematical Competencies, Spatial Abilities, and Mathematics Attitudes. Journal of Cognition and Development 2021, 22, 695-720.
28. Seethaler, P.M.; Fuchs, L.S. The Cognitive Correlates of Computational Estimation Skill Among Third-Grade Students. Learning Disabilities Research & Practice 2006, 21, 233-243.
29. Seethaler, P.M.; Fuchs, L.S.; Star, J.R.; Bryant, J. The cognitive predictors of computational skill with whole versus rational numbers: An exploratory study. Learning and Individual Differences 2011, 21, 536-542.
30. Simmons, F.R.; Willis, C.; Adams, A.-M. Different components of working memory have different relationships with different mathematical skills. Journal of experimental child psychology 2012, 111, 139-155.
31. Skagerlund, K.; Träff, U. Processing of space, time, and number contributes to mathematical abilities above and beyond domain-general cognitive abilities. Journal of Experimental Child Psychology 2016, 143, 85-101.
32. Soltani, A.; Mirhosseini, S. The contribution of general cognitive abilities and specific number skills toward arithmetic performance in students with mild intellectual disability. International Journal of Disability, Development and Education 2020, 67, 547-562.
33. Swanson, H.L. Cross-sectional and incremental changes in working memory and mathematical problem solving. Journal of Educational Psychology 2006, 98, 265.
34. Swanson, H.L.; Fung, W. Working memory components and problem-solving accuracy: Are there multiple pathways? Journal of Educational Psychology 2016, 108, 1153.
35. Swanson, L.; Kim, K. Working memory, short-term memory, and naming speed as predictors of children's mathematical performance. Intelligence 2007, 35, 151-168.
36. Träff, U. The contribution of general cognitive abilities and number abilities to different aspects of mathematics in children. Journal of experimental child psychology 2013, 116, 139-156.
37. Träff, U.; Olsson, L.; Skagerlund, K.; Östergren, R. Cognitive mechanisms underlying third graders' arithmetic skills: Expanding the pathways to mathematics model. Journal of Experimental Child Psychology 2018, 167, 369-387.
38. Vieira, F.D.; Ribeiro, D.O.; Farias, H.B.; Freitas, P.M. The Working Memory as Predictor of Performance in Arithmetic of Brazilian Students. Paidéia (Ribeirão Preto) 2021, 31.
39. Vukovic, R.K.; Kieffer, M.J.; Bailey, S.P.; Harari, R.R. Mathematics anxiety in young children: Concurrent and longitudinal associations with mathematical performance. Contemporary educational psychology 2013, 38, 1-10.
40. Vukovic, R.K.; Lesaux, N.K. The language of mathematics: Investigating the ways language counts for children's mathematical development. Journal of Experimental Child Psychology 2013, 115, 227-244.
41. Wong, T.T.Y. Is conditional reasoning related to mathematical problem solving? Developmental Science 2018, 21, e12644.
42. Xu, C.; Lafay, A.; Douglas, H.; Di Lonardo Burr, S.; LeFevre, J.-A.; Osana, H.P.; Skwarchuk, S.-L.; Wylie, J.; Simms, V.; Maloney, E.A. The role of mathematical language skills in arithmetic fluency and word-problem solving for first-and second-language learners. Journal of Educational Psychology 2022, 114, 513.
43. Xu, C.; LeFevre, J.-A.; Skwarchuk, S.-L.; Di Lonardo Burr, S.; Lafay, A.; Wylie, J.; Osana, H.P.; Douglas, H.; Maloney, E.A.; Simms, V. Individual differences in the development of children's arithmetic fluency from grades 2 to 3. Developmental Psychology 2021, 57, 1067.
44. Yang, X.; Yu, X. The relationship between mental rotation and arithmetic: do number line estimation, working memory, or place-value concept matter? British Journal of Educational Psychology 2021, 91, 793-810.

45. Zhang, X.; Räsänen, P.; Koponen, T.; Aunola, K.; Lerkkanen, M.-K.; Nurmi, J.-E. Knowing, applying, and reasoning about arithmetic: Roles of domain-general and numerical skills in multiple domains of arithmetic learning. Developmental Psychology 2017, 53, 2304.
46. Zheng, X.; Swanson, H.L.; Marcoulides, G.A. Working memory components as predictors of children's mathematical word problem solving. Journal of experimental child psychology 2011, 110, 481-498.
